# Suppression of kinesin family member-18A diminishes progression and induces apoptotic cell death of gemcitabine-resistant cholangiocarcinoma cells by modulating PI3K/Akt/mTOR and NF-κB pathways

**DOI:** 10.1371/journal.pone.0334147

**Published:** 2025-10-15

**Authors:** Pakornkiat Tanasuka, Phonpilas Thongpon, Sasitorn Chomwong, Suppakrit Kongsintaweesuk, Somchai Pinlaor, Thatsanapong Pongking, Sasithorn Watcharadetwittaya, Chawalit Pairojkul, Kitti Intuyod

**Affiliations:** 1 Department of Pathology, Faculty of Medicine, Khon Kaen University, Khon Kaen, Thailand; 2 Cholangiocarcinoma Research Institute, Khon Kaen University, Khon Kaen, Thailand; 3 Department of Parasitology, Faculty of Medicine, Khon Kaen University, Khon Kaen, Thailand; 4 Biomedical Sciences Program, Graduate School, Khon Kaen University, Khon Kaen, Thailand; 5 National Phenome Institute, Office of the President, Khon Kaen University, Khon Kaen, Thailand; The Hormel Institute (University of Minnesota), UNITED STATES OF AMERICA

## Abstract

Cholangiocarcinoma (CCA), particularly when associated with *Opisthorchis viverrini* infection, is often diagnosed at a late stage and exhibits high resistance to chemotherapy, notably gemcitabine. While kinesin family member 18A (KIF18A) is upregulated in opisthorchiasis-associated CCA, its precise function, especially in gemcitabine-resistant CCA, remains largely unexplored. Herein, expression of KIF18A in relation to survival and progression was assayed by TCGA database mining and immunohistochemistry of a tissue microarray derived from 84 CCA patients. For functional study, KIF18A was suppressed in gemcitabine-resistant CCA cells (KKU-213B^GemR^) using a CRISPR/Cas9 technique, followed by cellular and molecular analyses. Our results showed that KIF18A was highly expressed in CCA tissues compared to normal counterparts. Its expression was significantly correlated with tumor size and histological type of CCA but not with overall survival time. *In vitro*, KIF18A expression levels were increased in CCA cell lines, particularly KKU-213B^GemR^. Suppression of KIF18A significantly inhibited colony formation, migration and invasion by KKU-213B^GemR^ cells. In addition, KIF18A knockdown led to a significant increase in the sub-G1 population, indicating the occurrence of cellular apoptosis. Flow cytometry confirmed that suppression of KIF18A significantly induced early apoptotic cell death of KKU-213B^GemR^ cells. Suppression of KIF18A dramatically downregulated the expression of key oncogenic and survival signaling proteins, including PI3K (total and p-PI3K), Akt (total and p-Akt), mTOR (total and p-mTOR), NF-κB (total and p-NF-κB) and Bcl-2 in KKU-213B^GemR^ cells. Taken together, our findings suggest that KIF18A plays crucial roles in promoting the progression and survival of gemcitabine-resistant CCA cells, partly by modulating PI3K/Akt/mTOR and NF-κB pathways. Therefore, despite its lack of prognostic utility, KIF18A represents a promising therapeutic target for improving treatment outcomes in CCA patients, especially those who do not respond to gemcitabine treatment.

## Introduction

Cholangiocarcinoma (CCA) is a primary hepatobiliary malignancy that arises from cholangiocytes located in either intrahepatic, perihilar or extrahepatic bile ducts [1]. Although CCA used to be regarded as a rare cancer, its incidence is increasing over time and is particularly high in Southeast Asian countries and especially in northeastern Thailand, where the carcinogenic liver fluke (*Opisthorchis viverrini*) is endemic [1]. CCA is typically asymptomatic in its early stages, leading to late-stage diagnosis in most patients. As a result, surgical intervention is often not feasible, contributing to limited treatment options, poor prognosis and a short overall survival time [2]. Thus, systemic chemotherapy has been widely used for treatment of CCA patients. The combination of gemcitabine and cisplatin, with or without radiation therapy, is recommended as first-line regimen for treatment of CCA patients with advanced or inoperable disease [3]. However, chemoresistance significantly compromises the effectiveness of systemic chemotherapy for CCA, limiting therapeutic success [4]. Moreover, systemic chemotherapy usually produces adverse side effects, leading to diminishing quality of life for cancer patients [5]. Given these challenges, there is an urgent need to identify novel molecular targets for improving treatment efficacy and patient outcomes especially in those who do not respond to gemcitabine treatment.

Kinesin family member 18A (KIF18A) is a mitotic kinesin known to regulate chromosome alignment by controlling kinetochore–microtubule attachments and tension during cell division [6]. Overexpression of KIF18A has been documented in various malignancies associated with poor prognosis and survival, such as liver cancer [7], pancreatic cancer [8], esophageal cancer [9], glioblastoma [10], clear-cell renal cell carcinoma [11] and lung adenocarcinoma [12]. KIF18A has been reported to promote cancer cell proliferation, invasion, metastasis and treatment sensitivity of different cancer types [9,11,13–16]. A previous study reported that KIF18A is one of the most significantly overexpressed proteins in the plasma and tissues of opisthorchiasis-associated CCA, suggesting it may play a role in the development of this cancer [17]. Recently, small-molecule inhibition of KIF18A shows promising anti-cancer activity against chromosomally unstable tumors, a feature typically found in variety of cancer types including CCA [18,19]. Although roles of KIF18A in cancers and advances in development of KIF18A-targeting agents have been demonstrated, the biological function and mechanistic role of this protein in CCA, especially in gemcitabine-resistant CCA, remain largely unexplored.

In this study, we aimed to investigate the functional roles of KIF18A in CCA, particularly in gemcitabine-resistant CCA cells. In addition, the expression of KIF18A in CCA tissues and the association between its expression and clinicopathological features of CCA patients were also analyzed. The findings of this study suggest that KIF18A is a promising therapeutic target for CCA, especially in patients who are refractory to gemcitabine treatment.

## Materials and methods

### Determination of KIF18A expression in CCA tissues

The protocol in this study was approved by the Center for Ethics in Human Research at Khon Kaen University (HE671479). This study was conducted in accordance with the Declaration of Helsinki and the International Council for Harmonization (ICH) Good Clinical Practice Guidelines. Formalin-fixed, paraffin-embedded (FFPE) tissue samples from 84 CCA patients who underwent surgery at Srinagarind Hospital, Faculty of Medicine, Khon Kaen University, Khon Kaen, Thailand, and relevant clinicopathological data (initially accessed on 30 August 2024), were obtained from the Department of Pathology, Faculty of Medicine, Khon Kaen University, Thailand. Only data including age, gender, anatomical location of the tumor, tumor size, lymph node metastasis status, TNM stage, and histological type, were collected for use in this study. No information that could identify individual patients was obtained or reported in this study. Informed consent was waived by the ethics committee because anonymized data were used and the work did not involve direct contact with patients.

For public data analysis, the mRNA expression of KIF18A in CCA tissues (n = 36) compared with normal tissues (n = 9) was determined using GEPIA2 [20], a web server that utilizes data from The Cancer Genome Atlas (TCGA) database. For our study cohort, the expression of KIF18A protein in CCA tissues and adjacent non-cancerous tissues was explored using immunohistochemistry. In brief, CCA tissue-microarray slides (TMA) were constructed using an FFPE archive of 84 CCA patients. The core size was 4 mm and at least 4 cores were obtained for each patient. The TMA blocks were cut into 4 µm thickness and then subjected to deparaffinization, rehydration, and antigen retrieval through autoclaving with 1X Tris-EDTA buffer, pH 9.0. Then, slides were incubated with 3% H_2_O_2_ in methanol for 30 minutes and 5% FBS for 45 minutes to block endogenous peroxidase and non-specific binding sites, respectively. Subsequently, slides were incubated with rabbit anti-KIF18A (Cat. No. 19245-I-AP Proteintech, Rosemont, IL, USA) dilution 1:400 in 1% FBS overnight at 4°C. After washing with 1X PBS, pH 7.4, the slides were incubated with HRP-conjugated secondary antibody (Cat. No. 111-035-003 Jackson ImmunoResearch Laboratories Inc., West Grove, PA, USA), dilution 1:200, for 1 hour at room temperature. Immunoreactivity was visualized using 3,3-diaminobenzidine (DAB) (Merck, Darmstadt, Germany), and the slides were counterstained with Mayer’s hematoxylin. The expression level of KIF18A was graded as an H-score using QuPath software [21].

### Cell culture

An immortalized cholangiocyte cell line, MMNK-1 (JCRB1554) and CCA cell lines including KKU-055 (JCRB1551), KKU-100 (JCRB1568), and KKU-213B (JCRB1556) were sourced from the Japanese Collection of Research Bioresources Cell Bank (JCRB), National Institute of Biomedical Innovation, Japan. A gemcitabine-resistant KKU-213B (KKU-213B^GemR^) cell line was recently developed by our group as described previously [22]. All these cell lines were cultured in Dulbecco’s Modified Eagle’s Medium (DMEM; Gibco, Grand Island, NY, USA) supplemented with 10% FBS (Corning, NY, USA), 100 units/mL of penicillin, and 100 μg/mL of streptomycin (Gibco). All cell lines were grown at 37°C in a humidified incubator with 5% CO_2_.

### Reverse-transcription quantitative real-time polymerase chain reaction (RT-qPCR)

Total RNA was extracted from cell lines using the PureLink RNA Mini Kit reagent (Invitrogen, Carlsbad, CA, USA) and cDNA was synthesized using the RevertAid First Strand cDNA Synthesis Kit (Thermo Fisher Scientific, Waltham, MA, USA) following the manufacturer’s protocol. RT-qPCR was carried out in duplicate using FastStart Universal SYBR Green Master (Roche Applied Science, Mannheim, Germany) with the following sets of primers: *KIF18A* (5’-CAG TTCAGCCTATTCCTT-3’ and 5’-TATCACTGTTTGAGC-3’) and *GADPH* (5’-GTCTCCTCTGACTTCAACAGCG and 5’-ACCACCCTGTTGCTGTAGCCAA-3’). Reactions were performed using the Light Cycle 480 Real-Time PCR System (Roche). The mRNA expression levels of *KIF18A* relative to *GAPDH* were calculated using the 2^-ΔΔCt^ method [23].

### Western blot analysis

Total protein was extracted from cell lines using 1X RIPA buffer (Cell Signaling Technology, Danvers, MA, USA) supplemented with protease and phosphatase inhibitor cocktail (Cat. No.78440, Thermo Fisher Scientific, Waltham, MA, USA). The concentration of the extracted protein was quantified using the bicinchoninic acid assay (Thermo Fisher Scientific). Protein lysates (20 µg) were separated using SDS-PAGE and then transferred to Amersham Hybond-P PVDF membrane (GE HealthCare, Chicago, IL, USA). The membrane was blocked with BlockPRO 1 Min Protein-Free Blocking Buffer (Energenesis biomedical Co., Ltd, Taipei, Taiwan). After blocking of non-specific binding sites, the membrane was incubated overnight at 4°C with one of the following: KIF18A antibody at dilution 1:500 (Proteintech, Cat. No. 19245-I-AP), PI3K antibody at dilution 1:1000 (Cell Signaling Technology, Cat. No. 4249), p-PI3K at dilution 1:1000 (ABclonal Technology, Wuhan, China, Cat. No. AP0854), Akt at dilution 1:1000 (Cell Signaling Technology, Cat. No. 9272), p-Akt (193H12) at dilution 1:1000 (Cell Signaling Technology, Cat. No. 4058), mTOR at dilution 1:1000 (Cell Signaling Technology, Cat. No. 2983), p-mTOR at dilution at 1:1000 (Cell Signaling Technology, Cat. No. 5536), NF-κB at dilution 1:1000 (Santa Cruz Biotechnology, Cat. No. SC-8008), p-NF-κB at dilution 1:1000 (Cell Signaling Technology, Cat. No. 3033), Bcl-2 at dilution at 1:1000 (Abcam, Cambridge, UK, Cat. No. Ab7973), PARP at dilution 1:1000 (Cell Signaling Technology, Cat. No. 9542), cleaved PARP at dilution 1:1000 (Cell Signaling Technology, Cat. No. 5625) or β-Actin at dilution 1:2000 (Abcam, Cat. No. Ab3280). Afterward, the membrane was incubated with an appropriate HRP-conjugated secondary antibody at dilution 1:2000 (Jackson ImmunoResearch Laboratories Inc., Cat. No. 111-035-003). Proteins were detected using an enhanced chemiluminescence system (Merck) and the intensity of protein bands was quantified using ImageJ software (National Institutes of Health, MD, USA).

### CRISPR/Cas9 gene knockdown assay

A CRISPR/Cas9 all-in-one plasmid targeting KIF18A was obtained from GenScript Biotech Corporation (Piscataway, NJ, USA). Transfections were carried out using Lipofectamine 2000 (Thermo Fisher Scientific) following the manufacturer’s instructions. Briefly, 1 × 10^5^ of KKU-213B^GemR^ cells were seeded into 6-well plates and incubated for 24 hours. Lipofectamine-DNA complexes were prepared according to the manufacturer’s instructions and were then added to each well in serum-free growth medium. After a 6-hour incubation, the serum-free growth medium was replaced with antibiotic-free growth medium and cells were further incubated for either 24 or 48 hours before being returned to complete medium. Transfected cells were then harvested for further experiments and KIF18A expression levels were assessed via western blot analysis or RT-qPCR.

### Colony formation assay

A total of 1 × 10^3^ KKU-213B^GemR^ CCA cells from either negative control (NC), transfection control (TC), or KIF18A-knocked down (KIF18A-KD) groups were seeded into 6-well plates and incubated overnight. After adding the designated concentrations of gemcitabine (Fresenius Kabi India Pvt Ltd., Maharashtra, India) and incubation for 48 hours, the growth medium was replaced with normal growth medium and the cells were allowed to grow for up to 14 days. Then, medium was removed, and the cell were rinsed with 1X PBS, fixed with 6.0% glutaraldehyde, and stained with 0.5% crystal violet for 30 minutes at room temperature. Finally, the stained cells were rinsed with tap water and left to dry at room temperature. Images were taken and the number of colonies was counted using FIJI software [24].

### Wound-healing assay

A density of 8 × 10⁴ KKU-213B^GemR^ cells from either NC, TC, or KIF18A-KD groups were seeded in 24-well plates. Upon reaching approximately 80% confluence, a linear scratch was made across the cell monolayer using a sterile pipette tip to simulate a wound. Detached cells and debris were removed by washing the wells with 1X PBS. Immediately after scratching, the initial wound area was captured using an inverted microscope at 4X magnification. The migration of cells into the wound area was monitored and photographed at 12 hours post-treatment. The wound closure rate was subsequently calculated to evaluate cell migration capacity.

### Transwell cell migration and invasion assay

For cell invasion and cell migration assays, a Matrigel invasion chamber and Transwell chamber (Corning) were utilized, respectively. Cells transfected with KIF18A-CRISPR/Cas9 were trypsinized, counted in serum-free medium, and a total of 2 × 10^4^ cells in serum-free DMEM were seeded in the upper chamber. The lower chamber was filled with DMEM containing 20% FBS. After 48 hours, cells were washed with cold PBS, fixed with 4% paraformaldehyde, and stained with 1% crystal violet overnight. Then, adherent cells on the inner chamber surface were photographed, counted, and analyzed under an inverted phase-contrast microscope. Similar procedures were performed for the cell migration assay after KIF18A silencing, with 2 × 10^4^ cells cultured in a non-gel chamber.

### Flow cytometry analysis

The Annexin V-FITC/PI apoptosis detection kit (BioLegend, San Diego, CA, USA) was employed for the assessment of cell apoptosis following the manufacturer’s instructions. In brief, samples were treated with Annexin V-FITC and propidium iodide (PI) in the dark at room temperature for 15 minutes, and the analysis was carried out using a BD FACSCanto II flow cytometer (BD Biosciences, San Jose, CA, USA).

For cell-cycle analysis, cells were collected and fixed in 70% ethanol at −20°C overnight. Subsequently, cells were stained with FxCycle PI/RNase Staining Solution (Molecular Probes, Life Technologies, CA, USA) for 15 minutes at room temperature and the stained cell samples were analyzed using a BD FACSCanto II flow cytometer (BD Biosciences). Data were analyzed using BD FACS Diva software (BD Biosciences).

### Statistical analysis

The statistical analyses, including one-way ANOVA, Student’s t-test, and Kaplan-Meier survival analysis were carried out using Prism 9 software (GraphPad Software, Boston, MA, USA) and SPSS 28.0 software (IBM, Chicago, IL, USA). Most experiments were conducted using three independent biological replicates whereas western blot analysis was performed in two independent biological replicates. Any p value < 0.05 was considered statistically significant.

## Results

### KIF18A expression in CCA tissues with different clinicopathological characteristics

**Fig 1:**
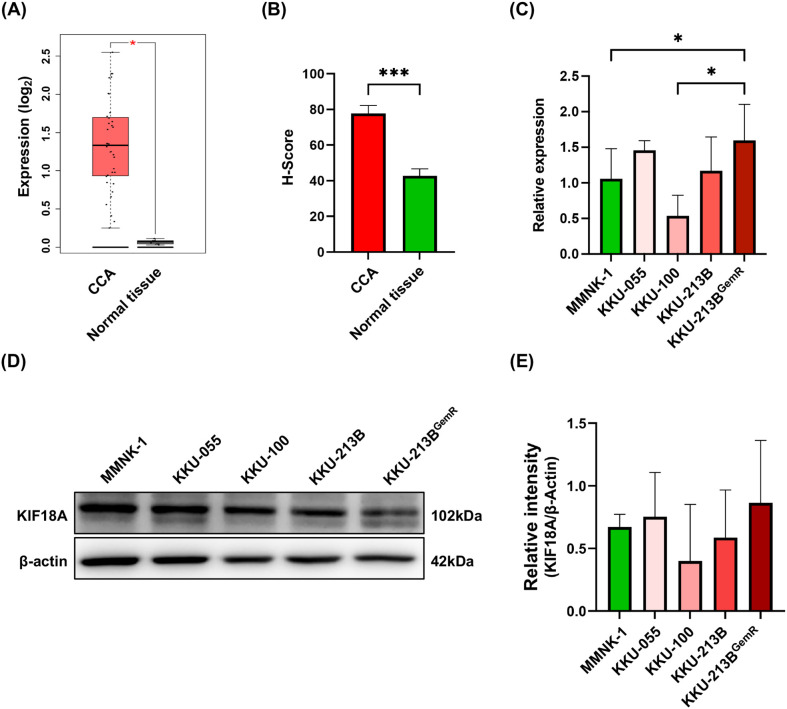
KIF18A expression analysis in CCA. **(A)** KIF18A mRNA expression levels in CCA tissues compared with normal tissues were analyzed using GEPIA2 database. **(B)** KIF18A protein expression levels in CCA tissues in comparison with normal adjacent tissues were determined using IHC. Expression levels are given as H-score. **(C, D)** Expression of KIF18A at transcriptional and translational levels in different CCA cell lines (KKU-055, KKU-100, KKU-213B, and KKU-213B^GemR^) and an immortalized cholangiocyte cell line (MMNK-1) was detected using RT-qPCR and western blotting, respectively. **(E)** Fold change representing the ratio between KIF18A and β-actin band intensity as determined by ImageJ. Data are presented as mean ± SD. * and *** indicated p < 0.05 and p < 0.001, respectively.

Firstly, we determined the expression of KIF18A in CCA tissues compared to normal adjacent tissues. Through the analysis of public database using GEPIA2, we observed that the mRNA expression levels of KIF18A in human CCA tissues (n = 36) was significantly higher (p < 0.05) than in normal adjacent tissues (n = 9) (Fig 1A). Next, we determined (using immunohistochemistry) the expression of KIF18A in a microarray we constructed with CCA tissues obtained from 84 CCA patients. KIF18A expression was observed in CCA tissues of all patients and was significantly higher in CCA tissues compared with normal bile ducts *(*p < 0.001) (Fig 1B). Expression levels of KIF18A in cancerous tissues from CCA patients with different clinicopathological features were analyzed as shown in Table 1. Although no statistical differences in KIF18A expression levels were observed across most of clinicopathological parameters, KIF18A expression was significantly higher in patients with a smaller tumor (89.53 ± 39.25 vs. 71.24 ± 39.36 for tumor size <7 cm and ≥7 cm, respectively; p = 0.043) and in CCA patients with different histological types (89.02 ± 40.25 vs. 68.55 ± 40.25 for papillary and non-papillary types, respectively; p = 0.018). Furthermore, KIF18A expression levels in CCA tissues, as observed in both the GEPIA2 database and our study cohort, were not significantly correlated with the overall survival time (OS) of CCA patients (S1 Fig). Thus, despite observing dysregulated KIF18A expression, its level did not demonstrate prognostic significance for CCA patients in this study cohort.

**Table 1:** Expression level of KIF18A in CCA patients with different clinicopathological features.

Clinicopathological parameters	Variable	No. of cases (n = 84)	KIF18A expression (Mean ±SD)	p-value
Age (Years)	<56	41	78.54* ± 40.91*	0.913
	>56	43	77.58* ± 39.18*	
Gender	Male	55	77.36* ± 40.16*	0.827
	Female	29	79.37* ± 39.77*	
Anatomical position	PC	36	77.62* ± 40.47*	0.933
	IC	48	78.37* ± 39.71*	
Tumor size	<7 cm.	36	89.53* ± 39.25*	0.043*
	≥7 cm.	43	71.24* ± 39.36*	
Lymph node metastasis	Yes	39	73.12* ± 39.79*	0.138
	No	38	86.58* ± 38.97*	
TNM stage	I-II	17	84.04* ± 38.71*	0.491
	III-IV	67	76.63* ± 40.21*	
Histological type	Papillary	39	89.02* ± 40.25*	0.018*
	Non-Papillary	45	68.55* ± 40.25*	

PC = Perihilar cholangiocarcinoma, IC = Intrahepatic cholangiocarcinoma.

### Expression of KIF18A in CCA cell lines

Next, we examined the KIF18A expression levels in CCA cell lines (KKU-055, KKU-100, KKU-213B and KKU-213B^GemR^) and an immortalized cholangiocyte cell line (MMNK-1). At the transcriptional level, the expression of KIF18A in KKU-213B^GemR^ was significantly higher than that in KKU-100 and MMNK-1 cell lines (Fig 1C; p < 0.05). The expression of KIF18A in the KKU-213B^GemR^ cell line also tended to be higher than that in the parental KKU-213B cell line. Western blotting showed that the levels of KIF18A protein in CCA cell lines and also in gemcitabine-resistant KKU-213B^GemR^ cells exhibited a similar pattern to that of transcriptional expression. However, the KIF18A protein expression levels in CCA cells as well as in KKU-213B^GemR^ cells, did not differ significantly from those in the MMNK-1 cell line (Fig 1D and 1E). Nevertheless, since gemcitabine resistance is one of the major problems in CCA and contributes to disease progression and poor prognosis, KKU-213B^GemR^ cells were used for the subsequent experiments.

### Knockdown of KIF18A changes cell morphology, reduces cell proliferation and suppresses migration and invasion by KKU-213B^GemR^ cells

**Fig 2:**
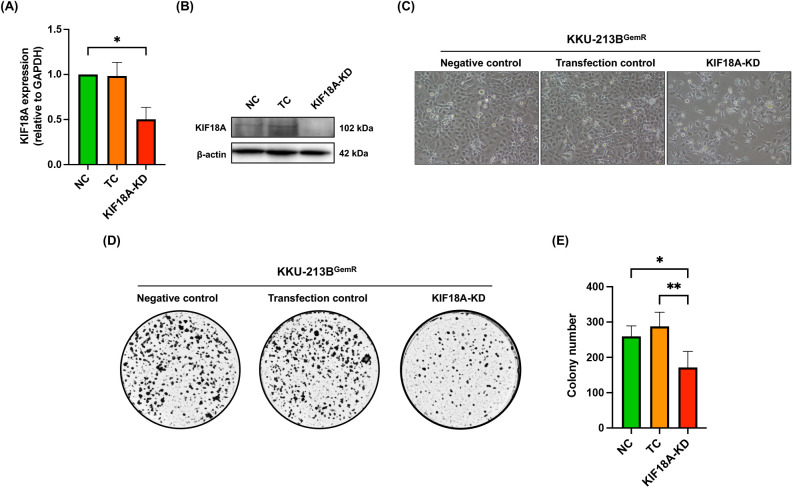
Effect of KIF18A knockdown on proliferation of KKU-213B^GemR^ CCA cells. Knockdown of KIF18A expression in the KKU-213B^GemR^ cell line was performed using CRISPR/Cas9. Expression of KIF18A after knockdown was determined using **(A)** qRT-PCR and **(B)** western blotting, respectively. **(C)** Morphological changes of KKU-213B^GemR^ cells following KIF18A knockdown compared with negative control and transfection control groups were observed using light microscopy. **(D, E)** Effect of KIF18A knockdown on proliferation of KKU-213B^GemR^ cells was determined using a clonogenic assay. **(E)** The number of colonies of KKU-213B^GemR^ cells in each group was counted using stereo microscope. Data are presented as mean ± SD. * and ** indicated p < 0.05 and p < 0.01, respectively. NC = Negative control; TC = Transfection control and KIF18A-KD = KIF18A knockdown.

**Fig 3:**
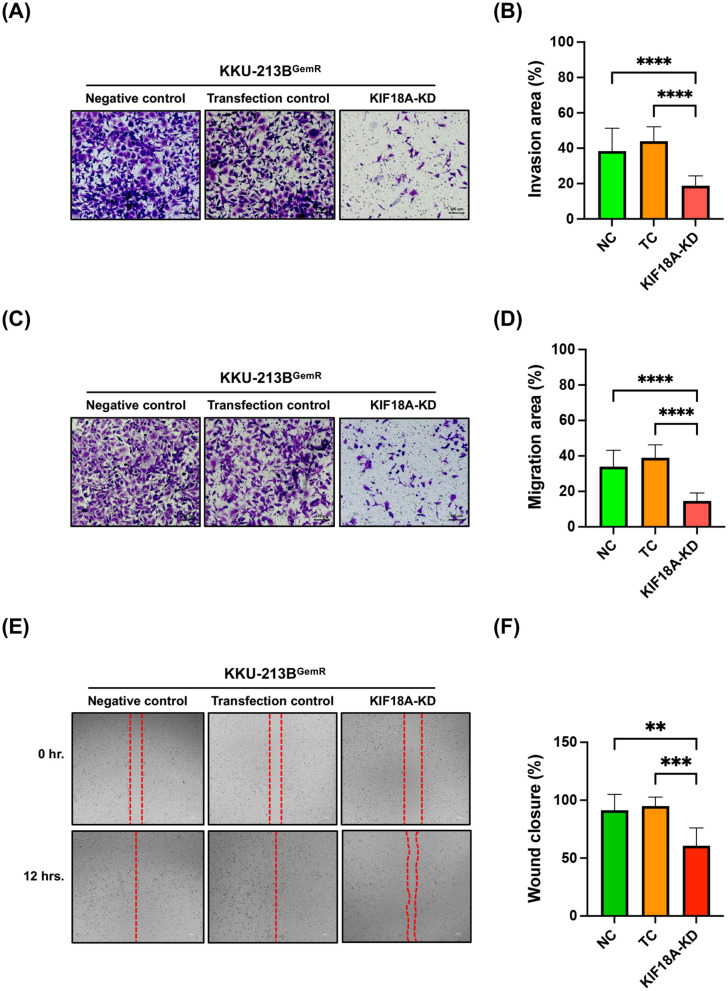
Effect of KIF18A knockdown on CCA cell invasion and migration. **(A, B)** Effect of KIF18A knockdown on CCA cell Matrigel invasion and **(C, D)** transwell migration capabilities. Graphs represent the extent of invasion and migration, respectively, by KKU-213B^GemR^ cells after KIF18A knockdown, measured at 48 hours after seeding. **(E, F)** Effect of KIF18A knockdown on CCA cell migration was also determined using the wound healing assay. Representative images of the wound healing assay in KKU-213B^GemR^ cells at 0 hour and 12 hours after seeding. Data are presented as mean ± SD. **, *** and **** indicated p < 0.01, p < 0.001 and p < 0.0001, respectively. NC = Negative control; TC = Transfection control and KIF18A-KD = KIF18A knockdown.

Although the expression levels of KIF18A protein were not obviously upregulated in CCA cells, including gemcitabine-resistant KKU-213B^GemR^ cells (Fig 1D and 1E), it remains to be clarified whether KIF18A is involved in cancer cell survival, progression, and gemcitabine resistance. To investigate the role of KIF18A in CCA cell proliferation, we knocked down KIF18A expression using the CRISPR/Cas9 technique (Fig 2A and 2B), which resulted in morphological changes, an indicator of cellular stress, and a significant reduction in cell proliferation (Fig 2C). This finding was further validated through a clonogenic assay, which demonstrated significantly lower colony formation in the KIF18A-KD group compared to both NC and TC groups (Fig 2D and 2E). These results indicate that KIF18A plays a critical role in promoting cancer cell proliferation in CCA. The roles of KIF18A in CCA cell invasion and migration were also investigated using Matrigel invasion and transwell migration assays. In comparison with the NC and TC groups, CCA cell migration was significantly lower in the KIF18A-KD group (Fig 3A and 3B). Similarly, transwell migration was significantly reduced in the KIF18A-KD group compared to the NC and TC (Fig 3C and 3D). Next, we conducted a wound-healing assay to support the role of KIF18A in KKU-213B^GemR^ CCA cell migration. The results showed that the percentage of wound closure in the KIF18A-KD group was lower than in the NC and TC groups (Fig 3E and 3F). Furthermore, to confirm whether KIF18A plays a role in gemcitabine resistance of CCA cells, KIF18A-knocked down KKU-213B^GemR^ cells were cultured with different concentrations of gemcitabine up to 72 hours and cell viability was assessed using the MTT assay. We found no significant difference in cell viability between KKU-213B^GemR^ cells in the KIF18A-KD group and control groups (NC and TC) when cultured in different concentrations of gemcitabine (S2 Fig). These results suggest that KIF18A is not significantly involved in gemcitabine resistance but has a role in cell viability and progression of KKU-213B^GemR^ cells.

### KIF18A knockdown induces cell cycle arrest and enhances apoptosis in KKU-213B^GemR^ cells

**Fig 4:**
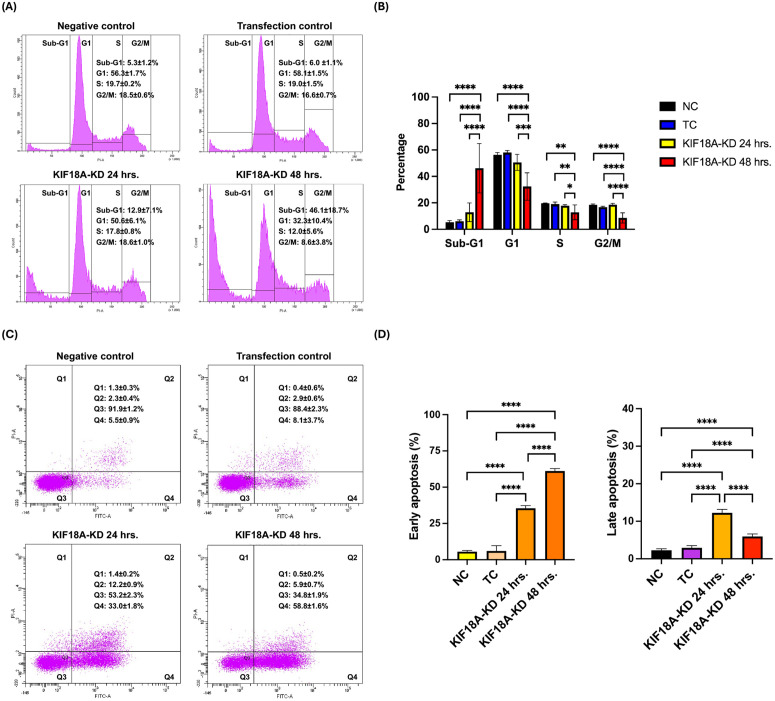
Effect of KIF18A knockdown on cell cycle and apoptosis of KKU-213B^GemR^ CCA cells. **(A)** Cell-cycle profile of KIF18A-knocked down KKU-213B^GemR^ cells compared with controls were analyzed by flow cytometry. **(B)** The percentage of cells from each experimental group in each cell-cycle phase is depicted and compared. **(C)** Representative flow cytometry histograms depict the pattern of Annexin V/PI staining of KKU-213B^GemR^ cells at 24-hour and 48-hour post-transfection. **(D)** The percentage of early and late apoptotic cells in each experimental group is depicted and compared. Data are presented as mean ± SD. *, **, *** and **** indicated p < 0.05, p < 0.01, p < 0.001 and p < 0.0001, respectively. NC = Negative control; TC = Transfection control and KIF18A-KD = KIF18A knockdown.

To investigate the roles of KIF18A in CCA cell proliferation and survival, we knocked down KIF18A expression in KKU-213B^GemR^ cells. Flow cytometry analysis was performed to assess changes in the cell-cycle distribution and apoptosis. Cell-cycle analysis revealed a significant increase in the Sub-G1 population in KIF18A-KD cells at 48-hour post-transfection compared to the NC and TC groups (Fig 4A and 4B). This suggests an increase in DNA fragmentation, indicative of apoptosis. Furthermore, at 48-hour post-transfection, the proportion of cells in the G1, S and G2/M phases was significantly reduced compared to the NC and TC groups, indicating that KIF18A depletion disrupts cell-cycle progression. To further confirm the impact of KIF18A knockdown on cell survival, Annexin V/PI staining was performed. Flow cytometry analysis demonstrated that the population of both early and late apoptotic cells was significantly increased in KIF18A-KD groups, at both 24- and 48-hour post-transfection, compared to the control groups (Fig 4C and 4D). This indicates that KIF18A knockdown promotes apoptosis of KKU-213B^GemR^ cells. Taken together, these findings suggest that KIF18A is crucial for CCA cell proliferation and survival, and its depletion induces cell-cycle arrest and apoptosis in KKU-213B^GemR^ cells.

### KIF18A suppression downregulates PI3K/Akt/mTOR and NF-κB pathways in KKU-213B^GemR^ cells

**Fig 5:**
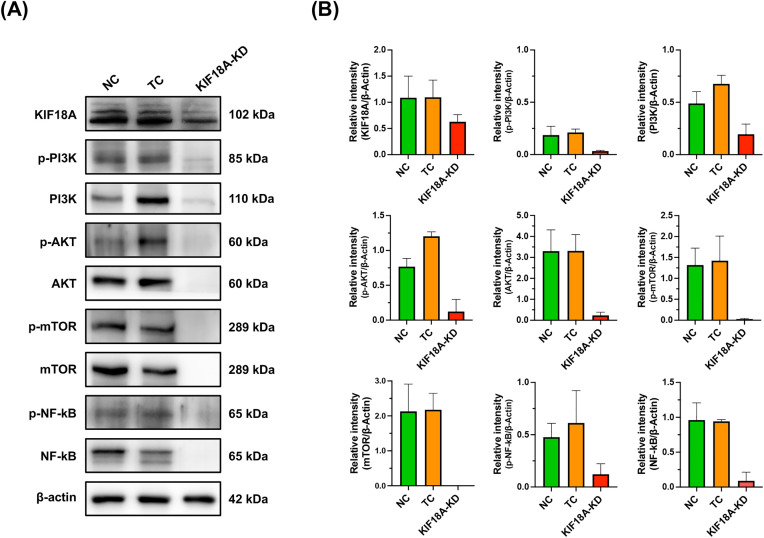
Effect of KIF18A knockdown on key signaling proteins in PI3K/Akt/mTOR and NF-κB pathways of KKU-213B^GemR^ CCA cells. **(A)** The levels of proteins involved in PI3K/Akt/mTOR and NF-kB pathways were measured using western blotting. Representative images show levels of KIF18A, PI3K, p-PI3K, Akt, p-Akt, mTOR, p-mTOR, NF-κB and p-NF-κB proteins in KIF18A-knocked down KKU-213B^GemR^ cells compared with controls. β-actin was used as a loading control. **(B)** The intensity of each protein band was measured using ImageJ. The expression levels of each protein are expressed as a relative intensity to that of β-actin. Data are presented as mean ± SD. NC = Negative control; TC = Transfection control and KIF18A-KD = KIF18A knockdown.

To explore the potential signaling pathways which underly the roles of KIF18A in proliferation, migration, invasion and survival of CCA cells, expression of KIF18A was inhibited in KKU-213B^GemR^ cells using the CRISPR/Cas9 technique. Western blot analysis was performed to detect the expression of several proteins, including KIF18A, PI3K, p-PI3K, Akt, p-Akt, mTOR, p-mTOR, NF-κB, p-NF-κB, and β-actin. The protein levels of KIF18A, PI3K, p-PI3K, Akt, p-Akt, mTOR, p-mTOR, NF-κB and p-NF-κB were dramatically reduced in the KIF18A-KD group compared to control groups (Fig 5). These results suggest that KIF18A modulates multiple signaling pathways, leading to promotion of the proliferation, survival, and progression of KKU-213B^GemR^ cells.

### KIF18A knockdown modulates the expression of apoptosis-related proteins in KKU-213B^GemR^ CCA cells

**Fig 6:**
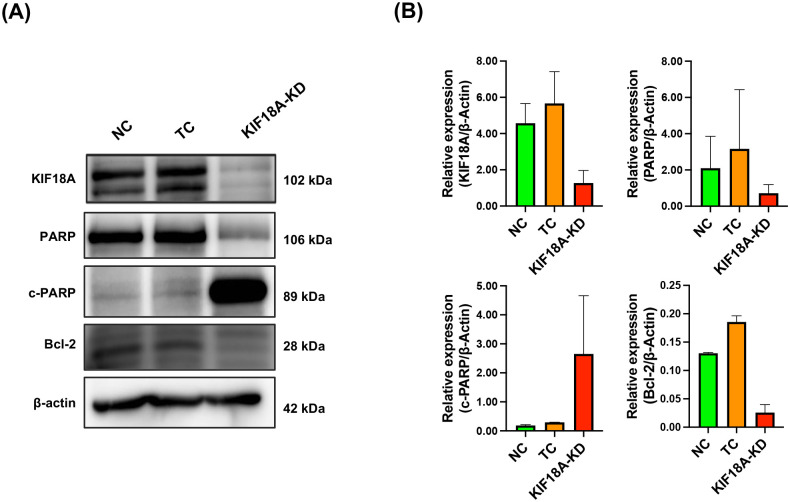
Effect of KIF18A knockdown on apoptosis-related proteins. **(A)** Representative images show levels of KIF18A, Bcl-2, PARP, and cleaved PARP (c-PARP) proteins measured by western blot in KIF18A-knocked down KKU-213B^GemR^ cells compared with controls. β-actin was used as a loading control. **(B)** The band intensity of each protein was measured using ImageJ. The expression level of each protein is expressed as intensity relative to that of β-actin. Data are presented as mean ± SD. NC = Negative control; TC = Transfection control and KIF18A-KD = KIF18A knockdown.

To further elucidate the role of KIF18A in apoptosis, we analyzed the expression levels of apoptosis-related proteins in KIF18A-KD cells using western blot analysis (Fig 6), which showed that KIF18A expression was reduced in the KIF18A-KD group compared to the NC and TC groups. Knockdown of KIF18A considerably increased the expression of the apoptotic marker, cleaved PARP (c-PARP). In contrast, the expression of the anti-apoptotic protein Bcl-2 was also decreased in KIF18A-KD cells. Quantification of protein levels confirmed these findings (bar graphs). These results suggest that depletion of KIF18A promotes apoptosis in KKU-213B^GemR^ cells in part by activating PARP cleavage and downregulating Bcl-2.

## Discussion

**Fig 7:**
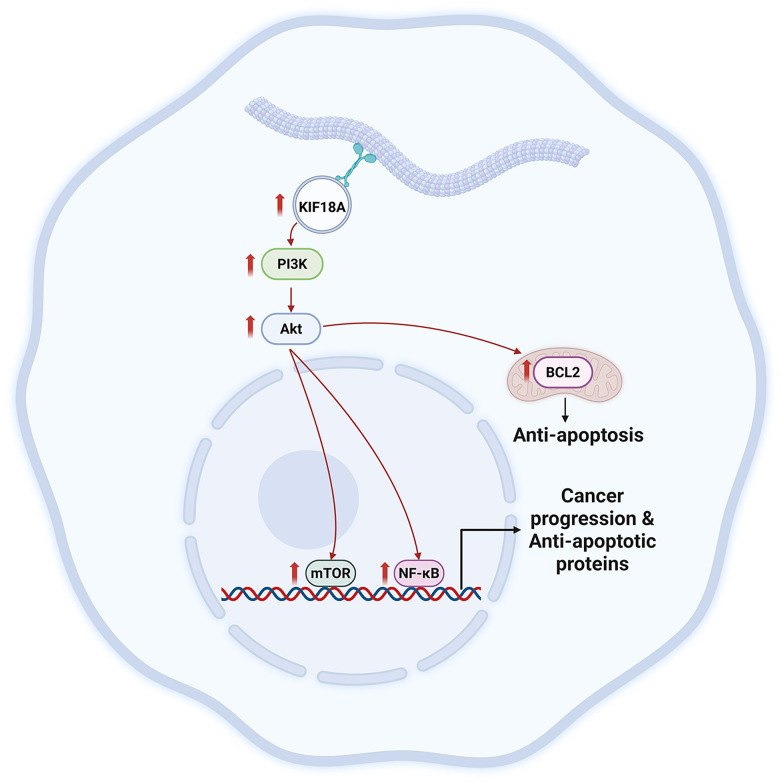
The proposed underlying mechanism for the roles of KIF18A in survival and progression of gemcitabine-resistant CCA cells. The image was created using BioRender (Agreement number TI28OK95X1).

Although KIF18A has been implicated previously in oncogenesis of various cancers, its functional role and mechanistic contribution to gemcitabine resistance of CCA have never been explored. In this study, we report for the first time that KIF18A has an oncogenic role in gemcitabine-resistant CCA cells by promoting cell proliferation, migration, invasion, and survival via modulation of the PI3K/Akt/mTOR and NF-κB signaling pathways. Based on our findings, the possible mechanism by which KIF18A contributes to survival and progression of gemcitabine-resistant CCA cells is shown in Fig 7. We suggest that KIF18A could be a potential therapeutic target for treatment of CCA.

Several lines of evidence demonstrate that KIF18A expression exhibits differential patterns across malignancies. In this study, our *in vitro* analysis revealed that the transcriptional expression level of *KIF18A* was significantly higher in KKU-213B^GemR^ cells compared to MMNK-1 cells while translational levels remained comparable. Conversely, KIF18A protein expression was markedly higher in CCA tissues relative to normal counterparts. These findings suggest that tumor microenvironment (TME) as well as extracellular matrix, which are present in CCA tissues but not in a cell culture system, might be involved in regulation of KIF18A expression. However, there are no direct studies or experimental validation to support this notion. Therefore, further work is required to reveal any role of TME and/or the extracellular matrix in regulation of KIF18A expression. Previous study has reported that KIF18A expression is regulated by the estrogen receptor alpha (ERα), indicating a role for estrogen [25]. ERα could bind to the KIF18A protein domain and be involved in the estrogen-mediated non-genomic cellular response [26]. This receptor is upregulated in CCA patients, both in cancerous tissues and CCA cell lines [27]. CYP19A1, an aromatase enzyme involved in estrogen synthesis, is also upregulated in CCA and significantly associated with poor prognosis of male CCA patients [27]. Furthermore, estrogen levels are elevated in male CCA patients and high serum estrogen levels are significantly associated with shorter OS of CCA patients [28]. Collectively, lack of TME as well as estrogen stimulation might underlie the low expression of KIF18A in our CCA cells.

We found that KIF18A expression was significantly elevated in CCA tissues compared to adjacent non-cancerous tissues. This is consistent with previous studies in various cancers [7–12]. KIF18A expression is significantly associated with key clinical and molecular features across multiple cancer types. These include prognosis, mutation status, DNA and RNA methylation patterns, TME, tumor mutational burden (TMB), microsatellite instability, mismatch repair genes, and drug sensitivity [29]. Moreover, KIF18A expression is associated with OS and disease-free survival in several cancer types. For instance, a KIF18A-based gene signature effectively predicts OS in patients with papillary renal-cell carcinoma, indicating its potential as both a prognostic biomarker and therapeutic target for renal-cell carcinoma [30]. In this study, we found that the expression of KIF18A was significantly different in CCA patients with different tumor sizes and histological types. However, no significant correlation was observed between KIF18A expression and OS in CCA patients. This discrepancy may be attributed to cancer-type-specific roles of KIF18A or differences in sample size, cancer biology, and clinical characteristics, as CCA is a highly heterogeneous cancer with diverse etiologies, anatomical subtypes, and a variable TME [31,32]. In addition, the prognostic impact of KIF18A may be masked by the influence of other dominant pathways driving cancer progression and survival, such as chronic inflammation [33,34] which is one of the key factors promoting development and progression of opisthorchiasis-associated CCA [35–37].

Although KIF18A did not show the prognostic significance for CCA patients in our cohort, our *in vitro* functional study found that KIF18A plays critical roles in the progression of CCA cells by promoting cancer cell proliferation, survival, migration, and invasion. This result is consistent with previous studies which reported that upregulation of KIF18A contributes to tumor progression in various malignancies, including breast cancer, lung cancer, liver cancer, colorectal cancer, and prostate cancer [15,38–42]. In addition to its role in cancer progression, KIF18A has also potential as a therapeutic target in CCA, similar to other cancer types [43,44]. We found that KIF18A knockdown led to sub-G1 cell-cycle arrest and significantly enhanced early apoptosis. This occurred concomitantly with upregulation of cleaved PARP and downregulation of anti-apoptotic protein Bcl-2. A previous study in lung adenocarcinoma similarly found that KIF18A knockdown induced cell-cycle arrest and cancer cell apoptosis [12].

Pathway analysis in our study revealed that KIF18A promoted cancer cell proliferation, survival, and progression (i.e., invasion and migration) of gemcitabine-resistant CCA cells through modulation of the PI3K/Akt/mTOR and NF-κB signaling pathways. These pathways are commonly implicated in promoting cell survival, proliferation, progression, and resistance to apoptosis in various cancers and also in CCA [45–49]. Previous studies reported that KIF18A regulates the phosphorylation but not the protein expression of PI3K and Akt in colorectal and breast cancer carcinogenesis models [50,51] and regulates progression of cervical squamous-cell carcinoma by enhancing phosphorylation of PI3K and Akt through upregulation of centrosome-associated protein E [52]. We found that suppression of KIF18A led to significant reduction in PI3K, Akt and mTOR protein levels, including both total and phosphorylated forms. Furthermore, downregulation of NF-κB was also observed upon KIF18A knockdown. In addition to regulation of NF-κB phosphorylation [53,54], the PI3K/Akt/mTOR signaling pathway also regulates NF-κB expression via CYR61 [55]. Therefore, apart from regulating the phosphorylation, KIF18A might also regulate the expression of PI3K/Akt/mTOR protein expression and hence, downregulation of these proteins led to suppression of NF-κB. However, the underlying mechanism by which KIF18A regulates PI3K/Akt/mTOR and NF-κB protein expression remains to be elucidated.

Although our findings clearly demonstrated that KIF18A promotes cancer cell proliferation, survival, migration, and invasion in gemcitabine-resistant CCA cells, its suppression did not enhance the sensitivity of these cells to gemcitabine treatment. This suggests that KIF18A is not directly involved in the mechanisms underlying chemoresistance. Gemcitabine resistance in CCA is commonly associated with alterations in drug uptake and efflux proteins, nucleotide metabolism, DNA damage repair, drug target, and apoptotic evasion [56]. However, KIF18A has never been implicated in these processes. In contrast, our findings demonstrated that KIF18A played central roles in enhancing tumor cell proliferation, migration, invasion, and survival, which are hallmark traits of cancer progression. These observations suggest that KIF18A primarily acts as a promoter of tumor aggressiveness rather than as a mediator of drug resistance. Additionally, we found that reduction by approximately 50% of KIF18A expression at both transcription and translation levels could significantly affect survival and progression of gemcitabine-resistant CCA cells. This reduction could significantly induce cellular apoptosis by approximately 70% at 48 hours, suggesting KIF18A as a potential therapeutic target for CCA patients who do not respond well to gemcitabine treatment. A small-molecule KIF18A inhibitor, VLS-1272, has been developed and is currently undergoing clinical trials. This compound demonstrates potent anticancer activity against cancer cells with high chromosomal instability but does not affect chromosomally stable cells such as normal cells [19]. Chromosome instability (CIN) is well recognized as an important factor that promotes cancer development, progression, and treatment resistance of several cancers [57–59]. Thus, with emphasizing the roles of KIF18A in CCA and the recent progress in development of a small-molecule inhibitor of KIF18A, these highlight the therapeutic potential of targeting KIF18A in aggressive cancers such as CCA with drug-resistant phenotype. Apart from targeting KIF18A as potential therapeutic strategy, there is recent evidence that upregulated KIF18A expression is positively associated with high TMB in pancreatic cancer [8]. Currently, immune checkpoint inhibitors including durvalumab and pembrolizumab have been approved for use in combination with gemcitabine and cisplatin for treatment of locally advanced or metastatic biliary-tract cancer [60–64]. Therefore, as TMB is well recognized as one of the biomarkers for predicting efficacy of immunotherapy [65,66], expression level of KIF18A might also be useful for predicting the feasibility of using immunotherapy for CCA patients. However, further study is required to confirm this speculation.

In conclusion, this study demonstrates that KIF18A is significantly overexpressed in CCA tissues and plays critical roles in promoting cancer cell proliferation, migration, invasion, and survival of gemcitabine-resistant CCA cells. KIF18A facilitates these oncogenic processes in part through the regulation of the PI3K/Akt/mTOR and NF-κB signaling pathways. Although KIF18A suppression did not enhance gemcitabine sensitivity, it effectively diminished tumor cell aggressiveness, underscoring its potential as a therapeutic target in drug-refractory CCA. However, this study has several limitations which should be addressed before the findings can be translated into clinical utility. First, the role of KIF18A in CCA progression was not assessed *in vivo*. Second, while our findings provide mechanistic insights, the precise molecular interactions between KIF18A and the PI3K/Akt/mTOR and NF-κB signaling pathways remain to be fully elucidated. Last, the prognostic value of KIF18A could not be confirmed in our cohort and may require further validation in larger, multicenter studies. Despite these limitations, our study provides evidence that targeting KIF18A may offer a novel strategy for effective treatment of CCA patients, especially those with gemcitabine resistance.

## Supporting information

S1 Fig Association of KIF18A expression and overall survival time of CCA patients. (A) KIF18A mRNA expression levels were retrieved from the GEPIA2 database and classified into low (n = 18) and high (n = 18) expression groups using the median value as cutoff. (B) KIF18A protein expression levels in a cohort of CCA patients (n = 84) was categorized into low- and high-expression groups using the median value of H-score retrieved from QuPath software as cutoff. Estimation of survival probability was performed using the Kaplan-Meier method, and the difference in survival time between groups was analyzed using a logrank test.
(TIF)

S2 Fig Effect of KIF18A knockdown on gemcitabine treatment. (A, B) The effect of KIF18A on gemcitabine sensitivity of KKU-213B^GemR^ was determined using MTT assay. KKU-213B^GemR^ cells were treated with different concentrations of gemcitabine for (A) 48 hours and (B) 72 hours. The cell viability was subsequently calculated relative to untreated controls in each group. All experiments were performed in three independent replicates. Data are presented as mean ± SD. NC = Negative control; TC = Transfection control and KIF18A-KD = KIF18A knockdown.
(TIF)

S3 Fig Original, uncropped western blot membranes. The original, uncropped membranes corresponding to Fig 5 and Fig 6 are shown. The experiments were performed in two independent biological replicates. NC = Negative control; TC = Transfection control and KIF18A-KD = KIF18A knockdown.
(PDF)

S4 File Raw data for plotting graphs. The data used for illustrating the graphs corresponding to Figs 1–6 are shown. Western blotting was performed in two independent biological replicates whereas all other experiments were performed in three independent biological replicates. NC = Negative control; TC = Transfection control and KIF18A-KD = KIF18A knockdown.
(PDF)
