# Supplementary figures and images for: Suppression of kinesin family member-18A diminishes progression and induces apoptotic cell death of gemcitabine-resistant cholangiocarcinoma cells by modulating PI3K/Akt/mTOR and NF-κB pathways
Source: PLoS One. 2025 Oct 15;20(10):e0334147. doi: 10.1371/journal.pone.0334147 (PMC12527176; doi:10.1371/journal.pone.0334147)

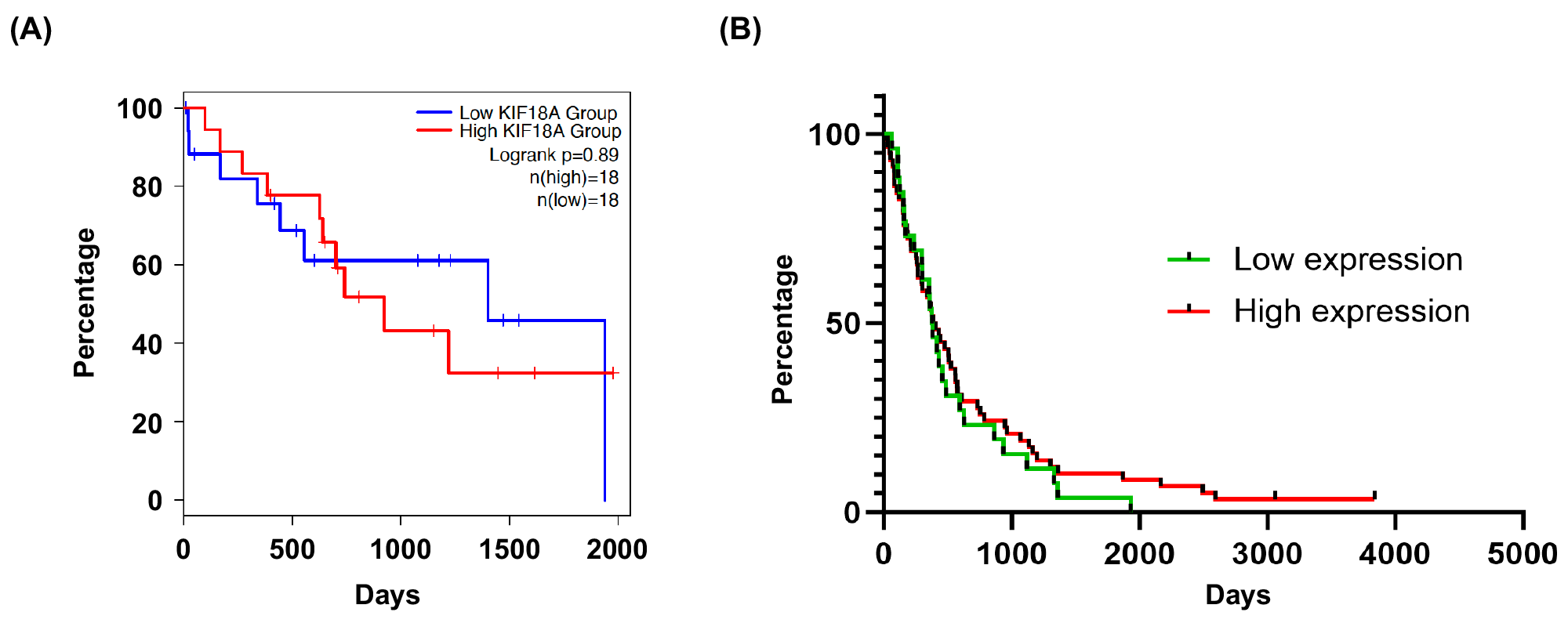

Supplement: S1 Fig — (A) KIF18A mRNA expression levels were retrieved from the GEPIA2 database and classified into low (n = 18) and high (n = 18) expression groups using the median value as cutoff. (B) KIF18A protein expression levels in a cohort of CCA patients (n = 84) was categorized into low- and high-expression groups using the median value of H-score retrieved from QuPath software as cutoff. Estimation of survival probability was performed using the Kaplan-Meier method, and the difference in survival time between groups was analyzed using a logrank test. (TIF) [file pone.0334147.s001.tif]

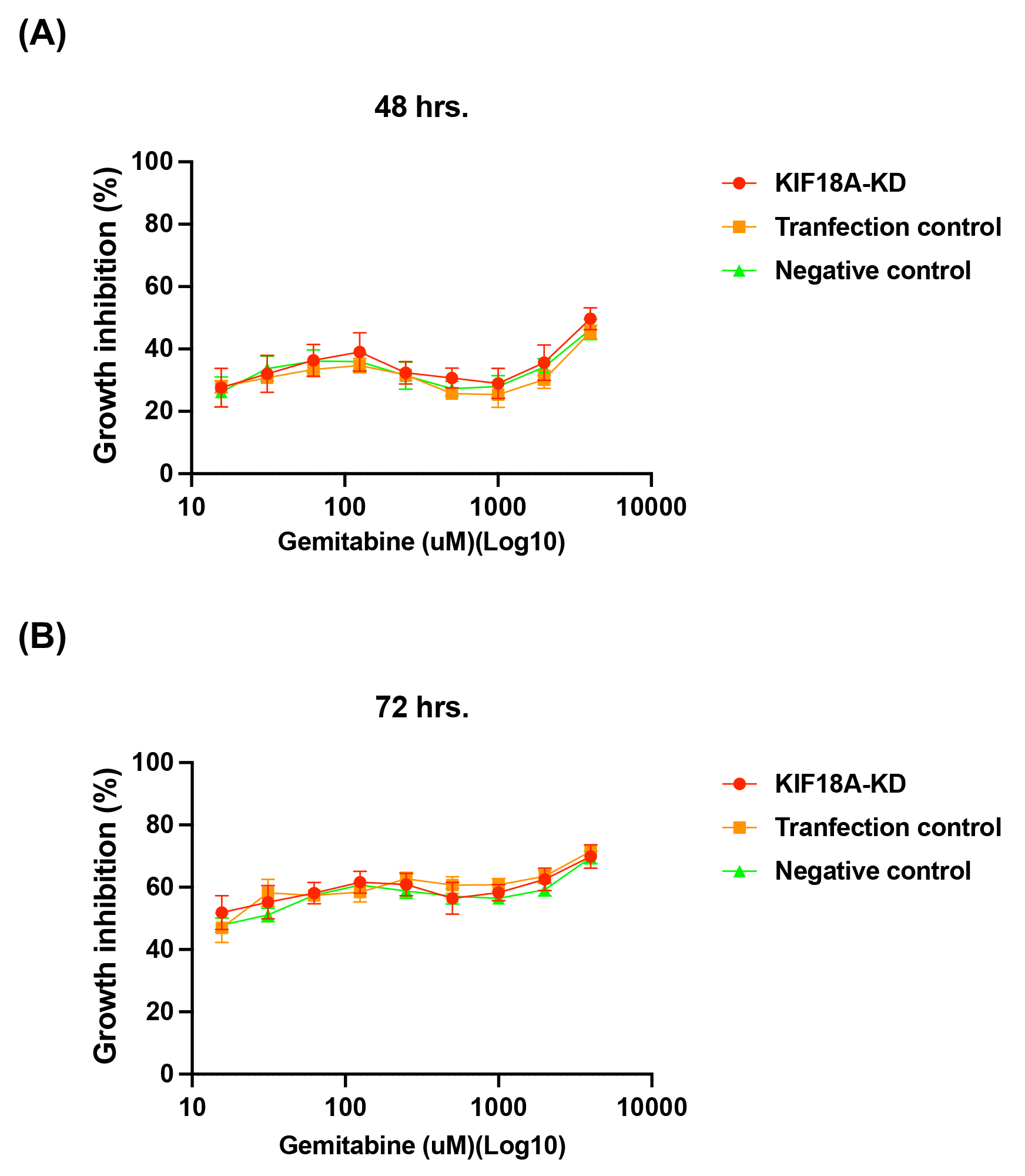

Supplement: S2 Fig — (A, B) The effect of KIF18A on gemcitabine sensitivity of KKU-213BGemR was determined using MTT assay. KKU-213BGemR cells were treated with different concentrations of gemcitabine for (A) 48 hours and (B) 72 hours. The cell viability was subsequently calculated relative to untreated controls in each group. All experiments were performed in three independent replicates. Data are presented as mean ± SD. NC = Negative control; TC = Transfection control and KIF18A-KD = KIF18A knockdown. (TIF) [file pone.0334147.s002.tif]

# Original, uncropped blot membranes for Fig.5

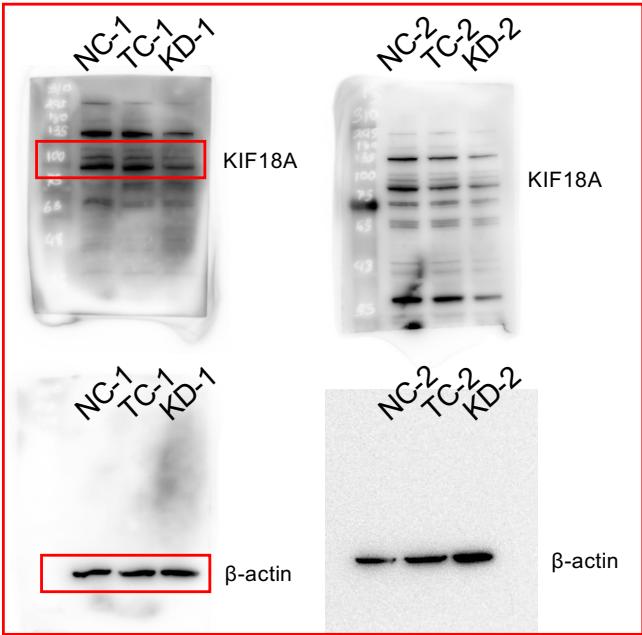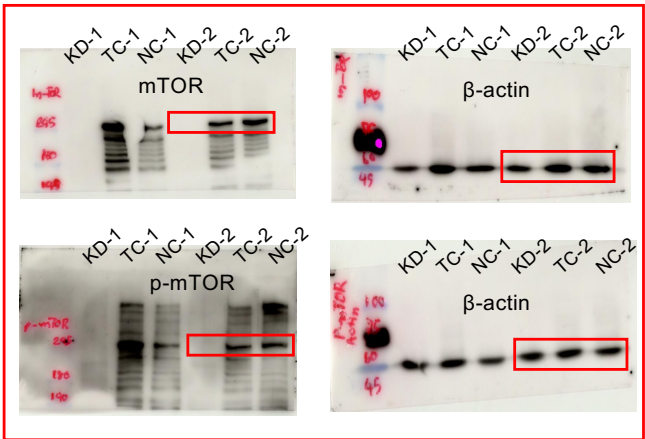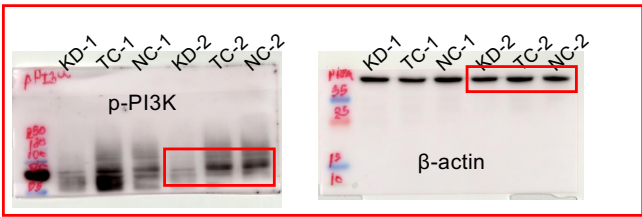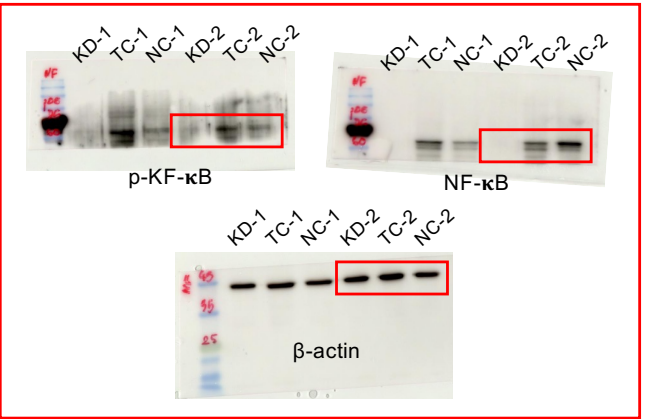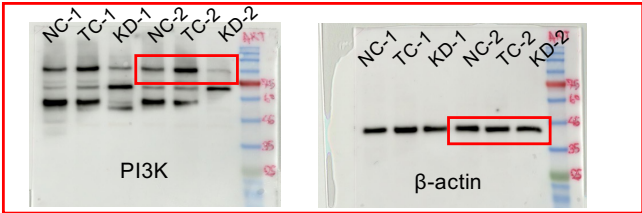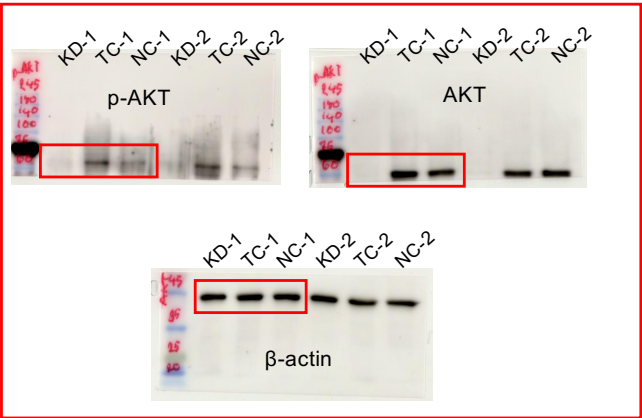

Original, uncropped blot membranes for Fig.6

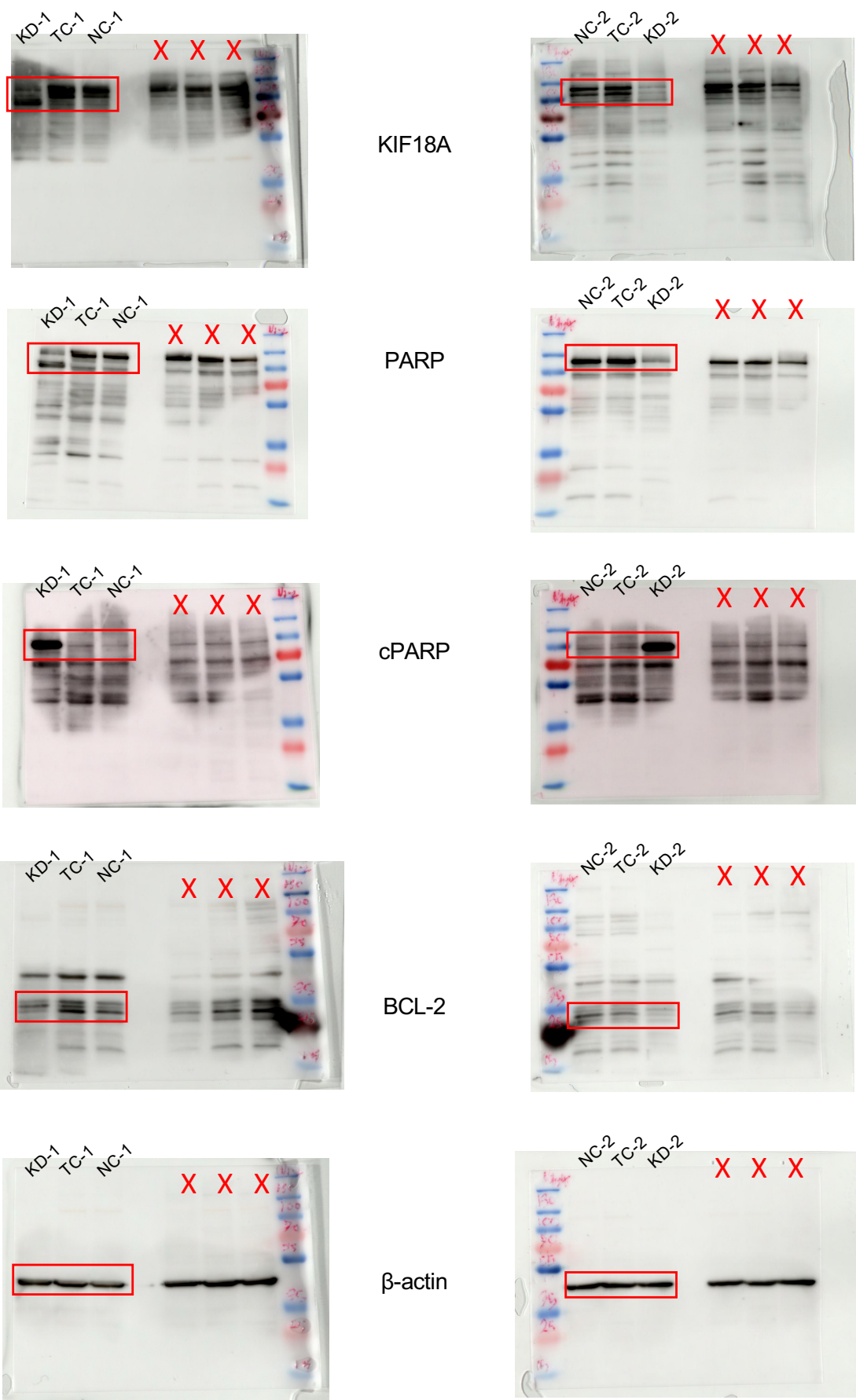

Supplement: S3 Fig — The original, uncropped membranes corresponding to Fig 5 and Fig 6 are shown. The experiments were performed in two independent biological replicates. NC = Negative control; TC = Transfection control and KIF18A-KD = KIF18A knockdown. (PDF) [file pone.0334147.s003.pdf]
