# Supplementary material for: Suppression of kinesin family member-18A diminishes progression and induces apoptotic cell death of gemcitabine-resistant cholangiocarcinoma cells by modulating PI3K/Akt/mTOR and NF-κB pathways
Source: PLoS One. 2025 Oct 15;20(10):e0334147. doi: 10.1371/journal.pone.0334147 (PMC12527176; doi:10.1371/journal.pone.0334147)
Supplement: S4 File — The data used for illustrating the graphs corresponding to Figs 1–6 are shown. Western blotting was performed in two independent biological replicates whereas all other experiments were performed in three independent biological replicates. NC = Negative control; TC = Transfection control and KIF18A-KD = KIF18A knockdown. (PDF) [file pone.0334147.s004.pdf]

Values used to illustrate graphs

Fig.1C. Relative KIF18A expression.

Sample	KIF18A mRNA expression				
	MMNK-1	KKU-055	KKU-100	KKU-2132B	KKU-213BGemR
N1	0.71	1.30	0.34	1.06	1.18
N2	0.93	1.53	0.40	0.76	1.45
N3	1.53	1.54	0.87	1.69	2.16
Mean	1.06	1.46	0.54	1.17	1.60
SD	0.42	0.14	0.29	0.47	0.51

Fig.1E. Relative KIF18A protein expression.

Sample	KIF18A relative intensity				
	MMNK-1	KKU-055	KKU-100	KKU-2132B	KKU-213BGemR
N1	0.71	0.71	0.92	1.02	1.43
N2	0.75	1.13	0.09	0.45	0.64
N3	0.56	0.42	0.19	0.30	0.52
Mean	0.67	0.75	0.40	0.59	0.86
SD	0.10	0.35	0.45	0.38	0.50

Values used to illustrate graphs

Fig.2A. Relative KIF18A expression after KIF18A knocked down.

	A	B	C	D
12	KIF18A	mRNA expression		
13		NC	TC	KIF18A-KD
14	N1	1	0.74	0.54
15	N2	1	0.95	0.26
16	N3	1	1.26	0.71
17	Mean	1.00	0.98	0.50
18	SD	0.00	0.26	0.23

Fig.2E. Number of colonies of KKU213B^{GemR} in different groups.

	A	B	C	D
1		Colony number		
2		NC	TC	KIF18A-KD
3	N1	302	339	128
4		272	301	114
5	N2	212	302	190
6		255	306	159
7	N3	248	235	228
8		267	244	209
9	Mean	259.33	287.83	171.33
10	SD	29.76	40.09	45.38

Values used to illustrate graphs

Fig.3B and 3D. Percentage of KKU213B^{GemR} cell invasion and migration in different groups.

% Cell invasion area

	NC	TC	KIF18A-KD
N1	31.70	49.82	25.96
	68.51	34.05	27.88
	31.50	34.82	23.03
	63.16	34.99	13.25
	32.68	43.14	21.71
	27.54	39.44	18.87
	29.23	29.16	19.69
	34.05	32.56	18.62
	29.40	40.21	23.57
	25.46	47.70	19.99
	31.19	42.85	22.82
	32.81	42.85	26.78
N2	33.73	48.44	26.04
	33.73	52.73	24.22
	61.16	44.35	17.96
	28.78	45.99	17.03
	30.38	48.85	16.84
	38.90	46.30	21.68
	48.99	47.44	13.43
	38.73	52.94	13.24
	36.55	36.82	16.94
	30.37	44.75	13.01
	44.18	41.59	13.99
	49.10	40.49	17.34
N3	25.90	39.67	28.13
	73.30	32.41	32.64
	31.28	63.94	9.32
	32.45	51.22	19.22
	35.12	57.53	11.38
	61.44	42.94	17.31
	37.75	61.36	14.35
	21.27	68.36	12.00
	28.96	49.13	15.89
	33.03	44.97	16.45
	36.34	37.95	12.45
	52.49	85.65	14.25
**Mean**	38.37	46.04	18.81
**SD**	12.94	11.11	5.56

% Cell migration area

	NC	TC	KIF18A-KD
N1	26.68	36.23	18.59
	23.16	38.48	18.13
	34.44	37.45	10.75
	26.15	33.50	13.75
	24.83	43.77	15.34
	27.38	33.42	15.42
	22.59	45.16	7.00
	33.79	36.83	16.22
	43.56	38.96	8.07
	40.32	42.00	20.23
	39.58	31.84	22.75
	42.05	32.09	11.37
N2	64.32	52.15	7.31
	29.24	54.30	13.66
	46.49	40.43	14.33
	39.38	32.67	25.99
	36.06	29.60	17.08
	21.60	32.51	16.40
	28.35	30.16	9.81
	29.67	43.93	14.81
	29.16	51.05	12.14
		47.24	16.62
		52.18	11.60
		53.82	19.95
N3	45.25	37.26	19.76
	31.39	31.15	16.75
	35.74	30.65	16.94
	25.73	34.21	16.67
	28.17	38.30	18.88
	42.99	30.45	14.74
	38.91	46.57	11.83
	35.27	34.01	9.42
	41.19	34.80	9.66
	18.85	41.64	6.98
	28.20	39.48	13.82
	39.11	34.85	14.48
**Mean**	33.93	38.98	14.65
**SD**	9.29	7.35	4.46

Values used to illustrate graphs

Fig.3F. Percentage of KKU213B^{GemR} wound closure in different groups.

	A	B	C	D	E	F	G	H	I	J
1		N1								
2		Uper well			Middle well			Lower well		
3		0 hr.	12 hrs	Wound closure (%)	0 hr.	12 hrs	Wound closure (%)	0 hr.	12 hrs	Wound closure (%)
4	NC	146586	39026.54	73.37636331	118955.4	19846.14	83.31632444	150265.6	0	100
5	TC	124868.8	0	100	113480.7	0	100	118595.1	0	100
6	KIF18A-KD	143920.7	67598.58	53.03068265	323735.1	123665	61.80056898	136351.6	67568.82	50.44517176
7										
8		N2								
9		Uper well			Middle well			Lower well		
10		0 hr.	12 hrs	Wound closure (%)	0 hr.	12 hrs	Wound closure (%)	0 hr.	12 hrs	Wound closure (%)
11	NC	91530.02	31510.71	65.57336383	70020.32	0	100	91530.02	0	100
12	TC	132151.2	0	100	142868.1	24214.18	83.05136784	152090.5	26585.79	82.51976239
13	KIF18A-KD	133539.9	82305.98	36.4363314	201246.5	109830.9	45.4247194	129485.9	35038.35	72.94040764
14										
15		N3								
16		Uper well			Middle well			Lower well		
17		0 hr.	12 hrs	Wound closure (%)	0 hr.	12 hrs	Wound closure (%)	0 hr.	12 hrs	Wound closure (%)
18	NC	123973.6	0	100	97785.64	0	100	104047.5	0	100
19	TC	182266.6	0	100	169177.4	19954.22	88.20514737	124485	0	100
20	KIF18A-KD	416499.5	81413.11	80.45301109	133993.4	34663.97	74.13008034	132400.3	34804.95	73.71232476

Values used to illustrate graphs

Fig.4B. Cell cycle profile of KKU213B^{GemR} in different groups.

	A	B	C	D	E	F	G	H	I	J	K	L	M	N	O	P	Q
1		NC								TC							
2		N1.1	N1.2	N2.1	N2.2	N3.1	N3.2	Mean	SD	N1.1	N1.2	N2.1	N2.2	N3.1	N3.2	Mean	SD
3	Sub-G1	6	3.7	4.1	7.1	5.7	5.4	5.333	1.26	7.9	5.3	4.7	6.5	5.4	6.3	6.0167	1.1392
4																	
5	G1	55.9	58.9	57.4	53.7	55.9	56	56.3	1.74	60.6	57.8	58.8	58.3	56.3	57	58.133	1.5042
6																	
7	S	19.6	19.8	19.4	19.8	19.9	19.9	19.73	0.2	16.2	20	19.4	18.6	20.6	19.5	19.05	1.5463
8																	
9	G2/M	18.4	17.5	19	19.3	18.4	18.4	18.5	0.62	15.2	16.8	17	16.5	17.5	17.1	16.683	0.7985

	R	S	T	U	V	W	X	Y	Z	AA	AB	AC	AD	AE	AF	AG
1	KIF18A-KD 24 hrs.								KIF18A-KD 48 hrs.							
2	N1.1	N1.2	N2.1	N2.2	N3.1	N3.2	Mean	SD	N1.1	N1.2	N2.1	N2.2	N3.1	N3.2	Mean	SD
3	4.8	14.7	22.4	12.6	4.7	18	12.867	7.1054	32.8	35.8	55.6	79.5	30.2	43.1	46.167	18.711
4																
5	58	46.8	42.6	51.7	57.1	47.5	50.617	6.1036	43.1	34.6	32.1	13.3	39.9	31	32.333	10.416
6																
7	17.6	18.4	17.5	17.3	19.2	16.9	17.817	0.8377	13.8	17.7	6.9	4.8	17.8	15.8	12.8	5.6182
8																
9	19.6	20	17.5	18.2	18.8	17.5	18.6	1.0564	10	11.8	5.4	2.4	12	10	8.6	3.8575

Values used to illustrate graphs

Fig.4D. Percentage of early and late apoptotic KKU213B^{GemR} cell death in different groups.

	NC								TC							
	N1.1	N1.2	N2.1	N2.2	N3.1	N3.2	Mean	SD	N1.1	N1.2	N2.1	N2.2	N3.1	N3.2	Mean	SD
Early Apoptosis	5.9	5.8	4.7	4.8	5	7	5.533	0.88	9.7	9.9	8.4	9.1	6.5	5.1	8.1167	1.9209
Late apoptosis	2.9	2.2	1.9	2	2.6	2	2.267	0.4	3.1	2.7	3.8	2.5	3.3	2.2	2.9333	0.582

KIF18A-KD 24 hrs.								KIF18A-KD 48 hrs.							
N1.1	N1.2	N2.1	N2.2	N3.1	N3.2	Mean	SD	N1.1	N1.2	N2.1	N2.2	N3.1	N3.2	Mean	SD
37.8	36.5	34.1	21.2	33.2	35.6	33.067	6.0417	61.9	47.2	60.5	63.6	59.2	60.5	58.817	5.8847
12.1	11.7	11	12.6	12.3	13.8	12.25	0.9397	5.3	6.2	4.9	6.5	6	6.7	5.9333	0.7005

Values used to illustrate graphs

Fig.5. Relative protein expression in KKU213B^{GemR} in different groups.

KIF18A	Relative intensity		
	NC	TC	KIF18A-KD
N1	0.79	0.86	0.53
N2	1.38	1.33	0.72
Mean	1.09	1.10	0.63
SD	0.41	0.33	0.14

pPI3K	Relative intensity		
	NC	TC	KIF18A-KD
N1	0.25	0.23	0.04
N2	0.13	0.19	0.03
Mean	0.19	0.21	0.03
SD	0.09	0.03	0.01

PI3K	Relative intensity		
	NC	TC	KIF18A-KD
N1	0.57	0.73	0.26
N2	0.41	0.62	0.13
Mean	0.49	0.68	0.19
SD	0.11	0.08	0.10

pAKT	Relative intensity		
	NC	TC	KIF18A-KD
N1	0.85	1.25	0.00
N2	0.68	1.16	0.25
Mean	0.77	1.20	0.12
SD	0.12	0.06	0.17

AKT	Relative intensity		
	NC	TC	KIF18A-KD
N1	2.58	2.76	0.34
N2	4.02	3.86	0.12
Mean	3.30	3.31	0.23
SD	1.02	0.78	0.16

pmTOR	Relative intensity		
	NC	TC	KIF18A-KD
N1	1.61	1.01	0.02
N2	1.03	1.84	0.04
Mean	1.32	1.42	0.03
SD	0.41	0.59	0.02

mTOR	Relative intensity		
	NC	TC	KIF18A-KD
N1	2.68	1.85	0.00
N2	1.57	2.51	0.00
Mean	2.13	2.18	0.00
SD	0.78	0.47	0.00

pNF-kB	Relative intensity		
	NC	TC	KIF18A-KD
N1	0.38	0.39	0.19
N2	0.57	0.83	0.05
Mean	0.48	0.61	0.12
SD	0.13	0.31	0.10

NF-kB	Relative intensity		
	NC	TC	KIF18A-KD
N1	1.13	0.96	0.18
N2	0.79	0.92	0.00
Mean	0.96	0.94	0.09
SD	0.25	0.02	0.13

Values used to illustrate graphs

Fig.6. Relative protein expression in KKU213B^{GemR} in different groups.

BCL-2	Relative intensity		
	NC	TC	KIF18A-KD
N1	0.13	0.19	0.02
N2	0.13	0.18	0.04
Mean	0.13	0.19	0.03
SD	0.00	0.01	0.01

PARP	Relative intensity		
	NC	TC	KIF18A-KD
N1	5.04	8.62	1.07
N2	2.43	3.82	1.35
Mean	3.74	6.22	1.21
SD	1.85	3.39	0.20

cPARP	Relative intensity		
	NC	TC	KIF18A-KD
N1	0.21	0.30	1.23
N2	0.17	0.29	4.07
Mean	0.19	0.29	2.65
SD	0.03	0.01	2.01

KIF18A-2	Relative intensity		
	NC	TC	KIF18A-KD
N1	5.34	6.90	1.76
N2	3.80	4.43	0.78
Mean	4.57	5.67	1.27
SD	1.09	1.75	0.69
